# Longitudinal Developmental Outcomes of Infants and Toddlers With Traumatic Brain Injury

**DOI:** 10.1001/jamanetworkopen.2022.51195

**Published:** 2023-01-17

**Authors:** Heather T. Keenan, Amy Clark, Rich Holubkov, Linda Ewing-Cobbs

**Affiliations:** 1 Division of Pediatric Critical Care, Department of Pediatrics, University of Utah School of Medicine, Salt Lake City; 2 Department of Pediatrics and Children’s Learning Institute, McGovern Medical School, University of Texas Health Science Center at Houston

## Abstract

**Importance:**

Among children, infants and toddlers have some of the highest rates of traumatic brain injury (TBI), but longitudinal information on their developmental outcomes to guide postinjury surveillance is sparse.

**Objective:**

To evaluate infants’ and toddlers’ development over 3 years following TBI compared with those with orthopedic injury (OI).

**Design, Setting, and Participants:**

A longitudinal observational cohort study was conducted at 2 level 1 pediatric trauma centers from January 20, 2013, to September 30, 2015; data analysis was performed from May 12 to October 20, 2021. Participants included children injured when younger than 31 months with TBI or OI who received emergency department care.

**Exposures:**

Mild, moderate, or severe TBI or OI.

**Main Outcomes and Measures:**

Parents completed baseline measures representing preinjury status and 3-, 12-, 24-, and 36-month postinjury status, using the Ages & Stages Questionnaire-3 (ASQ-3), with a mean reference value of 50 and higher scores indicating more advanced development. Linear mixed models characterized children’s outcomes for each ASQ-3 domain after adjustment for baseline ASQ-3, injury severity and group, age, injury mechanism (abuse or not), sex, prematurity, family function, social capital, and time. Interactions with time were evaluated.

**Results:**

Consent for participation was provided for 195 children; 184 parents (94%) completed a baseline survey. The cohort included 168 children who completed at least 1 follow-up survey: 48 (29%) mild; 54 (32%) complicated mild/moderate; 21 (13%) severe TBI; and 45 (27%) orthopedic injury. The cohort included 95 boys (57%), 49% injured before age 1 year; and 13% injured by abuse. Mean (SD) age at the time of injury was 13.9 (9.4) months. At 36 months, children with mild or complicated mild/moderate TBI performed similarly to children with OI across ASQ-3 domains. Children with severe TBI performed poorly as shown by negative mean differences in communication (–8.8; 95% CI, –13.8 to –3.8); gross motor (–10.1; 95% CI, –15.1 to –5.1); problem solving (–6.6; 95% CI, –11.2 to –1.9), and personal social (–6.3; 95% CI, –10.4 to –2.1) domains with little recovery over time. Children with abusive injury experienced decrements in ability over time in fine motor and personal social skills. Social capital was protective in communication (mean, 1.5; 95% CI, 0.3-2.7) and problem solving (mean, 1.2; 95% CI, 0.1-2.3) domains.

**Conclusions and Relevance:**

In this cohort study of children with TBI, children with severe injury showed little recovery. These findings suggest that early childhood intervention is needed, while children with milder injury remained on their developmental track and should continue routine developmental surveillance.

## Key Points

**Question:**

What is the recovery trajectory of children after traumatic brain injury (TBI) as an infant or toddler?

**Findings:**

In this cohort study of 168 children, compared with an orthopedic injury control group, children with mild TBI remained on their developmental track over a 3-year period. However, children with severe TBI showed little recovery after the first year postinjury and worsened in some skill areas.

**Meaning:**

The findings of this study suggest that children with severe TBI should receive early childhood intervention, while children with mild TBI should receive routine developmental surveillance.

## Teaser

This cohort study examines the developmental track of infants and toddlers over 3 years following traumatic brain injury.

## Introduction

Preschool-aged children have among the highest rates of pediatric traumatic brain injury (TBI),^1^ yet longitudinal information on their outcomes is sparse. Concern is high for very young children because skills in a rapid stage of development may be more vulnerable to the effects of injury.^2,3^ Even among very young children, infants may fare more poorly in some domains than toddlers after TBI.^4^

Studies of severe TBI among children injured as infants and toddlers show lower scores on tests of cognition and academic performance and less catch up than in older children.^2,3,5,6,7^ Severe TBI in this age group is complicated by the fact that nearly half of children younger than 2 years with severe TBI are injured by abuse.^8^ Children injured by abuse may differ in their outcomes from children with other severe TBI due to injury mechanics, time to care, and family environment.^9,10^

The few longer term outcome studies of very young children with mild TBI evaluated intelligence, behavior, and temperament. While children with mild TBI tend to score within the typical range in intelligence, behavior, and temperament, small differences exist in intelligence and behavior, especially when compared with typically developing children.^11,12,13,14^

However, few studies have assessed outcomes in longitudinal designs of young children with different mechanisms of injury that incorporate injury controls. To address this gap, we examined the trajectory of outcomes during the first 3 years after mild to severe TBI in a prospective, 3-year longitudinal cohort study of children injured prior to age 31 months with TBI. We hypothesized that children with severe TBI would show deficits and little recovery on the Ages & Stages screening tools,^15^ while children with mild TBI would perform similarly to those with orthopedic injury (OI).

## Methods

### Patient Population

Children younger than 31 months were recruited from 2 level 1 pediatric trauma centers: Primary Children’s Hospital in Salt Lake City, Utah, and the University of Texas Health Science Center at Houston/Children’s Memorial Hermann Hospital. This younger age group is reported separately from a larger cohort recruited up to age 15 years, because of the unique outcome measures used for very young children. English- and Spanish-speaking families presenting to the emergency department for children with TBI or OI were recruited and written informed consent was obtained from January 20, 2013, through September 30, 2015. Financial compensation was provided. Follow-up was completed in 2018. Data analysis for this study was conducted from May 12 to October 20, 2021. Children with severe developmental delay or very preterm birth (<32 weeks) were excluded. Institutional review board approval was obtained from both the University of Utah and the University of Texas Health Science Center at Houston. This study followed the Strengthening the Reporting of Observational Studies in Epidemiology (STROBE) reporting guideline for cohort studies.^16^

### Definitions

#### TBI Group

Traumatic brain injury was defined as an injury to the head with decreased level of consciousness, amnesia, and/or neuropsychological abnormality or diagnosed intracranial lesion.^17^ Traumatic brain injury severity was measured using the lowest emergency department pediatric Glasgow Coma Scale (GCS)^18^ and classified into mild (GCS≥13), moderate (GCS 9-12), and severe (GCS 3-8) categories. Mild TBI was defined as GCS 13 or higher on presentation to health care with GCS of 15 at discharge or after 24 hours if hospitalized, and 1 or more focal signs including a period of transient confusion, loss of consciousness for 30 minutes or less, irritability, vomiting, or lethargy, and/or transient neurologic abnormalities.^19,20^ Mild TBI was subclassified as complicated mild (cmild) based on intracranial hemorrhage diagnosed on computed tomography scan.^21^

#### Comparison Group

Children with an upper or lower extremity long bone fracture and no clinical signs or mechanism of injury suggestive of TBI were recruited contemporaneously with the TBI group. Comparison with an injury group isolates the effect of TBI from the effect of being injured and accounts for unmeasured preinjury differences. Injury severity was measured with the Abbreviated Injury Scale (level 1 denotes a minor injury, level 6 denotes an unsurvivable injury).^22^ Families were recruited sequentially on presentation to the hospital until the target of 40 children for each injury group and severity subgroup was reached.

Abusive head trauma was categorized by reviewing the child abuse team’s final consultation notes: 3 cases were adjudicated by the investigators as no conclusion was reported. Prematurity was defined as 32 to less than 37 weeks’ gestational age.

### Data Sources

Parents completed surveys of family demographic characteristics, family functioning and social support, and developmental outcomes as soon as possible after injury reflecting preinjury values. Demographic information included family composition, parent-identified race and ethnicity and child sex, parent-preferred language, income category, and health insurance status. Reporting of race and ethnicity was required by the funding agency; data are reported because it is important to know whether some communities have different impacts of injury. Follow-up assessments were collected at 3, 12, 24, and 36 months from injury. English-speaking families completed assessments in person, online, or by telephone. Bilingual study coordinators interviewed Spanish-speaking families in person or by telephone. Trauma registrars assigned Abbreviated Injury Scale scores. Computed tomography scans were performed at the treating clinician’s discretion and were read by each site’s pediatric neuroradiologists.

### Measures

#### Outcomes

The Ages & Stages Questionnaire-3 (ASQ-3) comprises parent-reported measures that assess communication, gross motor, fine motor, problem-solving, and personal social skills in children aged 1 to 60 months. Scores in a reference population have a mean (SD) of 50 (10). Higher scores indicate more advanced development. Children are categorized as appropriate, need to monitor (≥1 SD and <2 SD below the mean), and need to assess (≥2 SD below the mean corresponding to ≤ second percentile).^15^ Ages & Stages Questionnaire: Social-Emotional (ASQ:SE) measures 7 developmental and behavioral characteristics including self-regulation, compliance, communication, adaptive functioning, autonomy, affect, and interaction with others in children aged 3 to 66 months.^23^ The ASQ:SE is scored against an age-normed risk threshold. Higher scores indicate more problems. Sensitivity and specificity are favorable for identifying children who need evaluation for the ASQ-3 (0.86 and 0.85) and ASQ:SE (0.81 and 0.83). The ASQ-3 and ASQ-SE are available in both the English and Spanish languages.^24,25^

#### Family Environment

Families rated their preinjury function using the McMaster Family Assessment Device–General Functioning Scale.^26^ This scale includes 12 items scored from 1 to 4 with higher scores representing worse functioning. The Social Capital Index provides a total score measuring a person’s perceptions of personal, family, neighborhood, and spiritual community support, with higher scores representing more support.^27^ Family income relative to the 2013 federal poverty level was calculated using self-reported income category and family size.^28^

### Statistical Analysis

All children with evaluable outcomes preinjury and any follow-up time point were included in the analysis. Due to small numbers of children with moderate TBI, the moderate and complicated-mild groups were combined based on prior studies showing similar outcomes.^21^ Linear mixed models were used to characterize the association of the a priori selected variables of preinjury ASQ-3, injury severity and group, age, abuse, sex, family function, social capital, and time with each ASQ-3 domain. We centered time at 1 year of follow-up as the steepest recovery was expected to occur by this point. Interactions of each variable with time (considering up to a cubic relationship) were evaluated. Nonsignificant interactions (*P* ≥ .05) with time were trimmed from the model in an iterative fashion. Age was categorized into less than or greater than or equal to 1 year at time of injury. Age and abuse was evaluated as a single multilevel variable (<1 year, abuse; <1 year, no abuse; ≥1 year) because abuse occurred almost exclusively in children younger than 12 months. After the final multivariable model was determined, site, prematurity, and preferred language were entered into the model to assess whether they changed model estimates and should be included. We used casewise deletion for missing data. Outcome models were evaluated in SAS, version 9.4 (SAS Institute LLC) using PROC MIXED with an unstructured covariance matrix for the 4 time points (3, 12, 24, and 36 months); elements of this covariance matrix were estimated separately for the 3 age/abuse categories.

Post hoc analysis compared percentages of children by injury group and TBI severity who scored as developmentally appropriate vs needed monitoring or assessment (1 to ≥2 SD above mean) on the ASQ-3 using χ^2^ or Fisher exact test statistics. We calculated the proportion of children scoring in the typical range preinjury who were above the screening cutoff at the 3-year follow-up evaluation. The significance threshold with 2-sided testing was *P* < .05.

## Results

Informed consent was provided for 195 children, and 184 (94%) completed a preinjury assessment. The study cohort comprised 168 (91%) children who also completed at least 1 follow-up assessment (Figure); of these, 95 were boys (57%) and 73 were girls (43%); mean (SD) age at the time of injury was 13.9 (9.4) months. Of the 168 children, 160 (95%) completed a 12-month assessment and 134 (80%) completed a 36-month assessment. Preinjury assessments were completed a median of 7 (IQR, 3-13) days postinjury.

**Figure.**
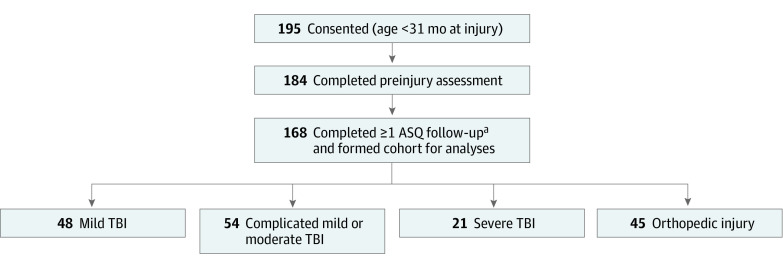
Cohort Flow Diagram ASQ indicates Ages & Stages Questionnaire. ^a^Assessments completed at each time point were 3 months (161 patients), 12 months (160 patients), 24 months (142 patients), and 36 months (134 patients).

The study cohort included 48 children with mild TBI (29%), 54 with complicated mild (n = 45) or moderate (n = 9) TBI (32%), 21 with severe TBI (13%), and 45 with OI (27%) (Table 1). The cohort was diverse with 49% racial and ethnic minority group representation, including 14% who preferred Spanish-language interviews. The most frequent mechanism of injury was fall (124 [74%]). Abuse was the injury mechanism in 22 children (13%), including half of the children with severe TBI. Preinjury, children were similar in family functioning, social capital, and income across injury groups. Ratings on the ASQ-3 were similar between children with TBI and OI, although children with severe TBI had lower mean preinjury ASQ-3 scores than other groups.

**Table 1.** Cohort Description^a^

Characteristic	Injury group				
	Mild TBI (n = 48)	Complicated mild/moderate TBI (n = 54)	Severe TBI (n = 21)	Orthopedic (n = 45)	Overall (n = 168)
**Child and injury characteristics**					
Enrollment site, Texas, No. (%)	29 (60)	25 (46)	5 (24)	20 (44)	79 (47)
Child sex, No. (%)					
Female	22 (46)	26 (48)	7 (33)	18 (40)	73 (43)
Male	26 (54)	28 (52)	14 (67)	27 (60)	95 (57)
Child race and ethnicity, No. (%)					
Black, non-Hispanic	9 (19)	4 (8)	1 (5)	3 (7)	17 (10)
Hispanic or Latino	16 (33)	14 (27)	6 (32)	14 (32)	50 (31)
White, non-Hispanic	21 (44)	26 (50)	10 (53)	26 (59)	83 (51)
Other, non-Hispanic^b^	2 (4)	8 (15)	2 (11)	1 (2)	13 (8)
Age at time of injury, mean (SD), mo	13.0 (9.0)	10.3 (8.6)	12.0 (10.2)	20.2 (7.5)	13.9 (9.4)
Age at time of abuse, No. (%)					
<1 y, abuse	2 (4)	7 (13)	11 (52)	1 (2)	21 (13)
<1 y, no abuse	24 (50)	29 (54)	2 (10)	6 (13)	61 (36)
≥1 y	22 (46)	18 (33)	8 (38)	38 (84)	86 (51)
Premature (<37 wk), No. (%)	4 (8)	7 (13)	1 (5)	4 (9)	16 (10)
Injury mechanism, No. (%)					
Assault	2 (4)	8 (15)	11 (52)	1 (2)	22 (13)
Pedestrian or bicycle	0	2 (4)	1 (5)	2 (4)	5 (3)
Motorized vehicle	3 (6)	1 (2)	4 (19)	0	8 (5)
Fall	40 (83)	42 (78)	3 (14)	39 (87)	124 (74)
Struck by or against	2 (4)	0	0	2 (4)	4 (2)
Other	1 (2)	1 (2)	2 (10)	1 (2)	5 (3)
ED GCS Total, median (IQR)^c^^,^^d^	15.0 (15.0-15.0)	15.0 (15.0-15.0)	3.0 (3.0-6.0)	NA	15.0 (14.0-15.0)
Head and neck AIS, median (IQR)^e^	2.0 (1.0-2.0)	3.0 (2.0-4.0)	4.0 (3.0-4.0)	0.0 (0.0-0.0)	2.0 (0.0-3.0)
Maximum AIS score, excluding head, median (IQR)^e^	0.0 (0.0-1.0)	1.0 (0.0-1.0)	1.0 (0.0-2.0)	2.0 (2.0-3.0)	1.0 (0.0-2.0)
**Preinjury family characteristics**					
Preferred language: Spanish, No. (%)	8 (17)	7 (13)	1 (5)	8 (18)	24 (14)
Married, No. (%)	26 (55)	35 (69)	14 (67)	33 (73)	108 (64)
McMaster family functioning, mean (SD)^f^	1.4 (0.4)	1.5 (0.5)	1.4 (0.5)	1.4 (0.5)	1.4 (0.5)
Social Capital Index, mean (SD)^g^	3.6 (1.1)	3.4 (1.3)	3.9 (1.0)	3.5 (1.2)	3.5 (1.2)
Income relative to poverty level, mean (SD)	2.1 (1.7)	2.2 (1.6)	2.7 (1.7)	2.1 (1.4)	2.2 (1.6)
Baseline ASQ scores, mean (SD)^h^					
Communication	48.8 (12.4)	44.4 (13.9)	41.0 (16.5)	47.5 (12.5)	46.0 (13.6)
Gross motor	51.4 (11.2)	49.2 (12.1)	43.8 (15.3)	51.1 (14.2)	49.6 (13.0)
Fine motor	47.2 (12.8)	46.7 (12.9)	40.2 (19.2)	47.4 (11.9)	46.2 (13.6)
Problem solving	49.2 (9.2)	46.5 (12.7)	36.0 (19.3)	48.0 (11.8)	46.3 (13.1)
Personal-social	48.2 (10.2)	46.4 (12.3)	46.1 (11.8)	46.8 (11.9)	47.0 (11.5)

Abbreviations: AIS, Abbreviated Injury Scale; ASQ, Ages & Stages Questionnaire; ED, emergency department; GCS, Glasgow Coma Scale; NA, not applicable; TBI, traumatic brain injury.

^a^ Missing values were as follows: race and ethnicity (n = 5), married (n = 4), Social Capital Index (n = 2), and income (n = 13).

^b^ Other races reported were American Indian or Alaska Native (n = 2), Asian (n = 3), Native Hawaiian or other Pacific Islander (n = 2), and multiple races (n = 6).

^c^ There were 15 children with severe TBI who had received a muscle relaxant at the time ED GCS was performed.

^d^ Mild TBI (GCS≥13), moderate (GCS 9-12), and severe (GCS 3-8).

^e^ Level 1 denotes a minor injury; level 6 denotes an unsurvivable injury.

^f^ Contains 12 items scored 1 to 4 (possible range, 4-48), with higher scores reflecting worse function.

^g^ Possible range is 1 to 5, with higher scores indicating more social capital.

^h^ With a mean reference value of 50, higher ASQ scores indicate more advanced development.

Table 2 reports unadjusted means and mean differences in ASQ scores by injury group and time period for each outcome. Children with severe TBI had substantively worse scores in each domain at each time point compared with other groups, although mean change from 12 to 36 months (5.3; 95% CI, –5.1 to 15.7) was not significant. Unadjusted trajectories by age and abuse status demonstrate lack of return to baseline for children with abuse on average compared with other groups. eTable 1 in Supplement 1 details severe TBI by age and abuse status, and eFigure 1 and eFigure 2 in Supplement 1 show unadjusted mean ASQ-3 outcome scores over time.

**Table 2.** ASQ Outcomes at Preinjury and 12, 24, and 36 Months Postinjury^a^

ASQ outcome	Mean score				Change over time, mean (95% CI) score			
	Preinjury (n = 168)	12 mo (n = 160)	24 mo (n = 142)	36 mo (n = 134)	Preinjury to 12 mo (n = 160)	Preinjury to 24 mo (n = 142)	Preinjury to 36 mo (n = 134)	12-36 mo Postinjury (n = 133)
Communication								
Mild TBI	48.8	48.6	53.7	53.5	–1.1 (–5.5 to 3.3)	4.5 (0.9 to 8.1)	5.4 (0.1 to 10.6)	5.8 (2.5 to 9.0)
cMild/moderate TBI	44.4	43.4	52.9	51.7	–0.3 (–4.9 to 4.3)	9.6 (5.1 to 14.2)	8.2 (3.4 to 13)	9.1 (4.3 to 13.9)
Severe TBI	41.0	31.5	41.5	37.0	–8.5 (–19.1 to 2.1)	–3.5 (–16.1 to 9.2)	–6.3 (–20.4 to 7.8)	5.3 (–5.1 to 15.7)
Orthopedic injury	47.5	48.9	54.3	55.8	1.0 (–4.7 to 6.7)	6.8 (3.1 to 10.5)	9.2 (4.2 to 14.2)	5.2 (1.2 to 9.2)
Gross motor								
Mild TBI	51.4	55.0	54.8	55.6	3.8 (0.2 to 7.3)	3.3 (–0.8 to 7.4)	3.7 (–0.3 to 7.7)	1.3 (–1.4 to 4.0)
cMild/moderate TBI	49.2	53.6	54.5	53.1	5.0 (1.7 to 8.3)	6.2 (3 to 9.5)	5.1 (1.8 to 8.4)	–0.1 (–2.5 to 2.2)
Severe TBI	43.8	32.1	39.2	34.7	–10.9 (–19.2 to –2.6)	–5 (–18.6 to 8.6)	–9.0 (–20.5 to 2.5)	4.9 (–4.6 to 14.3)
Orthopedic injury	51.1	53.8	54.2	56.3	2.8 (–0.9 to 6.6)	2.5 (–1.9 to 6.9)	4.0 (–0.6 to 8.7)	2.1 (–0.8 to 4.9)
Fine Motor								
Mild TBI	47.2	45.6	38.8	44.6	–1.9 (–6.7 to 3)	–7.7 (–14.2 to –1.2)	–1.5 (–8.4 to 5.4)	–0.5 (–6.6 to 5.5)
cMild/moderate TBI	46.7	46.4	39.5	47.8	0.3 (–4.8 to 5.4)	–6.1 (–12.3 to 0.2)	2.2 (–3.9 to 8.4)	3.1 (–2.9 to 9.0)
Severe TBI	40.2	31.0	33.1	31.5	–9.3 (–19.2 to 0.7)	–5.4 (–19 to 8.2)	–9.5 (–21.3 to 2.3)	1.8 (–5.4 to 9.1)
Orthopedic injury	47.4	40.1	46.5	50.4	–7.8 (–13.5 to –2.2)	–1.7 (–5.2 to 1.9)	2.2 (–2.8 to 7.3)	11.0 (3.3 to 18.7)
Problem solving								
Mild TBI	49.2	48.2	50.7	53.5	–0.8 (–4.4 to 2.8)	1.3 (–3.1 to 5.8)	5.0 (0.2 to 9.8)	6.2 (2.4 to 10.0)
cMild/moderate TBI	46.5	46.4	52.5	53.3	0.4 (–4.8 to 5.6)	7.1 (3 to 11.2)	7.7 (3.1 to 12.4)	8.3 (3.5 to 13.1)
Severe TBI	36.0	32.0	41.9	40.7	–3.5 (–11.9 to 4.9)	5.0 (–9.2 to 19.2)	1.3 (–11.3 to 14)	8.7 (–0.6 to 17.9)
Orthopedic injury	48.0	49.7	53.2	55.9	1.1 (–3.1 to 5.3)	4.7 (0.5 to 8.8)	8 (4.5 to 11.5)	5.3 (2.0 to 8.6)
Personal social								
Mild TBI	48.2	51.2	53.0	53.2	3.0 (–0.9 to 6.9)	5.0 (1.1 to 8.9)	5.8 (1.4 to 10.1)	2.5 (0.1 to 4.9)
cMild/moderate TBI	46.4	49.4	51.6	51.5	3.6 (–0.6 to 7.9)	6.4 (1.8 to 11.1)	7 (2.7 to 11.4)	3.0 (0.0 to 5.9)
Severe TBI	46.1	34.0	39.2	39.7	–11.4 (–20.9 to –1.9)	–7.7 (–24 to 8.6)	–7.5 (–20.3 to 5.2)	6.0 (–2.9 to 14.9)
Orthopedic injury	46.8	51.1	51.8	55.4	4.0 (0.5 to 7.4)	4.3 (0.4 to 8.1)	8.1 (4.6 to 11.7)	4.6 (1.5 to 7.6)

Abbreviations: ASQ, Ages & Stages Questionnaire; cMild, complicated mild; TBI, traumatic brain injury.

^a^ With a mean reference value of 50, higher ASQ scores indicate more advanced development.

In the mixed-effects models (Table 3 and Table 4), adjusting for preinjury scores and other covariates, examination of group effects with the OI group as the referent indicated that children with mild and complicated mild (cmild)/moderate TBI performed similarly to children with OI at both 12 and 36 months after injury in all domains: communication (mild: mean difference, –1.6; 95% CI, –4.8 to 1.6; cmild/moderate: mean difference, –0.8; 95% CI, –4.0 to 2.5), gross motor (mild: mean difference, –0.6; 95% CI, –3.6 to 2.4; cmild/moderate: mean difference, –0.3; 95% CI, –3.2 to 2.7), fine motor (mild: mean difference, –2.3; 95% CI, –6.5 to 1.9; cmild/moderate: mean difference, 0.8; 95% CI, –3.5 to 5.0), problem-solving (mild: mean difference, –1.0; 95% CI, –3.9 to 2.0; cmild/moderate: mean difference, 0.2; 95% CI, –2.9 to 3.3), and personal social (mild: mean difference, –0.8; 95% CI, –3.4 to 1.8; cmild/moderate: mean, difference 0.5; 95% CI, –2.3 to 3.2) domains. We report 12- and 36-month outcomes for the adjusted analyses as injury group and time did not interact.

**Table 3.** ASQ-3 Communication, Gross Motor, and Fine Motor Outcomes at 1 and 3 Years Following Injury^a^^,^^b^

Characteristic	ASQ-3 score, mean (95% CI)					
	Communication^c^		Gross motor		Fine motor	
	12 mo	36 mo	12 mo	36 mo	12 mo	36 mo
Injury group						
Mild TBI	−1.6 (−4.8 to 1.6)	NC	−0.6 (−3.6 to 2.4)	NC	−2.3 (−6.5 to 1.9)	NC
cMild/moderate TBI	−0.8 (−4.0 to 2.5)	NC	−0.3 (−3.2 to 2.7)	NC	0.8 (−3.5 to 5.0)	NC
Severe TBI	−8.8 (−13.8 to −3.8)	NC	−10.1 (−15.1 to −5.1)	NC	−4.7 (−11.4 to 1.9)	NC
Orthopedic injury	[Reference]	[Reference]	[Reference]	[Reference]	[Reference]	[Reference]
Female	1.7 (−0.9 to 4.2)	NC	3.5 (1.4 to 5.5)	NC	3.1 (0.02 to 6.2)	NC
Premature birth	−3.5 (−8.2 to 1.2)	NC	−4.4 (−8.0 to −0.9)	NC	−5.6 (−10.8 to −0.4)	NC
Age at injury/abuse						
< 1 y, Abuse	−15.4 (−25.0 to −5.8)	−12.3 (−23.5 to −1.1)	−9.0 (−18.4 to 0.3)	−6.3 (−16.3 to 3.7)	−0.7 (−10.0 to 8.6)	−22.0 (−32.6 to −11.3)
< 1 y, No abuse	−1.4 (−6.2 to 3.4)	−0.1 (−3.9 to 3.7)	1.7 (−1.4 to 4.8)	1.0 (−1.7 to 3.6)	8.1 (3.2 to 13.1)	−7.1 (−12.2 to −2.0)
> 1 y	[Reference]	[Reference]	[Reference]	[Reference]	[Reference]	[Reference]
Family function (1-point increase)	1.1 (−2.0 to 4.2)	NC	−0.8 (−3.2 to 1.6)	NC	−0.3 (−4.0 to 3.4)	NC
Social Capital Index (1-point increase)	1.5 (0.3 to 2.7)	NC	−0.5 (−1.7 to 0.8)	0.2 (−0.9 to 1.3)	1.3 (−0.1 to 2.7)	NC

Abbreviations: ASQ-3, Ages & Stages Questionnaire-3; cMild, complicated mild; NC, no change in the estimate from 12 to 36 months; TBI, traumatic brain injury.

^a^ Model estimates reflect estimated mean differences between the specified subgroup and the reference group for categorical variables. Model estimates for family function and social capital reflect the mean change in outcomes for a 1-point increase in the score.

^b^ Model estimates that differ across time points (eg, age at injury/abuse) reflect results that varied over time due to an interaction between the main outcome and time. Those that are constant across time (eg, injury group) reflect no interaction between the main outcome and time. The eAppendix in Supplement 1 provides details on main outcomes and interactions with time included in each outcome model.

^c^ Example interpretation for ASQ-3 Communication: children with severe TBI scored a mean of −8.8 points lower than children with orthopedic injury at 12 and 36 months following injury. Child sex and premature birth were not statistically significant. There was an interaction with time and age at injury/abuse. Abused children younger than 1 year at the time of abuse had an additional decrement of 15.4 points at 12 months and 12.3 points at 36 months compared with children who were 1 year or older. Family function was not statistically important to the model.

**Table 4.** ASQ-3 Problem Solving and Personal Social Outcomes at 1 and 3 Years Following Injury^a^^,^^b^

Characteristic	ASQ-3 score, mean (95% CI)			
	Problem solving		Personal social	
	12 mos	36 mos	12 mos	36 mos
Injury group				
Mild TBI	−1.0 (−3.9 to 2.0)	NC	−0.8 (−3.4 to 1.8)	NC
cMild/moderate TBI	0.2 (−2.9 to 3.3)	NC	0.5 (−2.3 to 3.2)	NC
Severe TBI	−6.6 (−11.2 to −1.9)	NC	−6.3 (−10.4 to −2.1)	NC
Orthopedic injury	[Reference]	[Reference]	[Reference]	[Reference]
Female	1.4 (−1.0 to 3.8)	NC	1.2 (−0.9 to 3.3)	NC
Premature birth	−2.0 (−7.8 to 3.8)	−1.6 (−6.7 to 3.4)	−2.8 (−7.1 to 1.4)	2.0 (−2.2 to 6.3)
Age at injury/abuse				
< 1 y, Abuse	−11.3 (−19.2 to −3.4)	−12.0 (−22.6 to −1.5)	−9.2 (−18.1 to −0.2)	−16.2 (−27.0 to −5.4)
< 1 y, No abuse	−4.9 (−9.3 to −0.6)	−1.5 (−5.0 to 2.0)	−1.4 (−4.1 to 1.3)	−0.9 (−3.7 to 1.8)
> 1 y	[Reference]	[Reference]	[Reference]	[Reference]
Family function (1-point increase)	0.9 (−2.0 to 3.7)	NC	−0.4 (−2.9 to 2.0)	NC
Social Capital Index (1-point increase)	1.2 (0.1 to 2.3)	NC	0.5 (−0.4 to 1.5)	NC

Abbreviations: ASQ-3, Ages & Stages Questionnaire-3; cMild, complicated mild; NC, no change in the estimate from 12 to 36 months; TBI, traumatic brain injury.

^a^ Model estimates reflect estimated mean differences between the specified subgroup and the reference group for categorical variables. Model estimates for family function and social capital reflect the mean change in outcomes for a 1-point increase in the score.

^b^ Model estimates that differ across time points (eg, age at injury/abuse) reflect results that varied over time due to an interaction between the main outcome and time. Those that are constant across time (eg, injury group) reflect no interaction between the main outcome and time. The eAppendix in Supplement 1 provides details on main outcomes and interactions with time included in each outcome model.

The severely injured TBI group had marked deficits across ASQ-3 domains relative to the OI group in communication (mean difference, –8.8; 95% CI, –13.8 to –3.8), gross motor (mean difference, –10.1; 95% CI, –15.1 to –5.1), problem-solving (mean difference, –6.6; 95% CI, –11.2 to –1.9), and personal social (mean difference, –6.3; 95% CI, –10.4 to –2.1) domains. The severe TBI group remained impaired compared with the OI group over the 3-year follow-up period as shown by the lack of a group × time interaction. Children younger than 1 year with abusive injury had an additional decrement in their outcomes with little recovery. Children with abusive injury scored more poorly in communication (mean difference, –15.4; 95% CI, –25.0 to –5.8), problem-solving (mean difference, –11.3; 95% CI, –19.2 to –3.4), and personal social (mean difference, –9.2; 95% CI, –18.1 to –0.2) domains at 12 months than older children. Interactions with time revealed that, at 36 months, the abuse group worsened over time in fine motor (mean difference, –22.0; 95% CI, –32.6 to –11.3) and personal social (mean difference, –16.2; 95% CI, –27.0 to –5.4) skills. Children who were injured before age 1 year who were not abused had worse fine motor skills at 36 months (mean difference, –7.1; 95% CI, –12.2 to –2.0) compared with older children. For the ASQ-3 gross motor domain, male sex (female: mean difference, 3.5; 95% CI, 1.4-5.5) and prematurity (mean difference, –4.4; 95% CI, –8.0 to –0.9) were risk factors. Male sex (female: mean difference, 3.1; 95% CI, 0.02-6.2) and prematurity (mean difference, –5.6; 95% CI, –10.8 to –0.4) were also risk factors for lower fine motor scores. Preinjury family function did not impact children’s recovery; however, family social capital seemed important in both the communication (mean difference, 1.5; 95% CI, 0.3-2.7) and problem-solving (mean difference, 1.2; 95% CI, 0.1-2.3) domains.

Children with mild TBI on average scored slightly lower than children with OI across all domains; however, these differences were not statistically significant. In post hoc analysis, we found that children in the mild TBI group had a similar percentage of children greater than 1 SD below the mean at 24 and 36 months for ASQ-3 communications, gross motor, and personal social domains. However, a higher percentage of children with mild TBI scored greater than 1 SD below the mean in fine motor (26% vs 9%; *P* = .05) and problem solving (13% vs 0%; *P* = .06) domains at 36 months.

The ASQ:SE status was examined by the change in ASQ:SE scores between baseline and 36 months compared with the screening cutoff indicating the need for more in-depth evaluation. Injury group was significantly associated with a new positive screen, with nearly half of children in the severe TBI group reporting a new problem. The mild, cmild, and moderate TBI groups were similar to the OI group (eTable 2 in Supplement 1).

## Discussion

In this longitudinal cohort study, we examined the trajectory of young children’s developmental status as measured by the ASQ-3 and ASQ:SE for 3 years after a TBI compared with those with an OI. We found that children with severe TBI had persisting and at times worsening deficits across multiple domains while children with less severe injury appeared to stay on their developmental track. In addition to severe TBI, risk factors for poorer outcomes included abusive injury and, for fine motor skills, younger age at TBI. Male sex and prematurity were risk factors in motor and fine motor domains. Although preinjury family functioning was not significantly related to ASQ-3 outcomes, family social capital was associated with communication and problem-solving outcomes.

Children with severe TBI had substantial deficits and showed little to no recovery over the 3-year follow-up period in all domains compared with children with OI. Children with abusive injuries fared most poorly and acquired additional deficits over time in fine motor, personal social, and socioemotional domains. These results add to prior findings of very young children with severe TBI.^3,6,11^ Anderson et al^3^ studied 8 infants with severe TBI and found global IQs in the low average range that worsened from 12- to 30-month follow-up, in contrast to older children with severe TBI and infants with mild TBI. In contrast, Crowe et al^11^ found that, while children with nonabusive moderate/severe TBI injured at a mean age of 21 months had full-scale IQs below those of typically developing controls, they were still within the reference range in cognitive function at 3.5 and 7 years postinjury. Ewing-Cobbs and colleagues^6^ reported that children injured prior to age 71 months with moderate or severe injury who were able to be tested on a standardized assessment scored well below typically developing children with no improvements over time on a measure of intelligence. Our cohort, similar to that of Ewing-Cobbs et al,^6^ showed little to no recovery. Results of this study emphasize the persistence of cognitive, motor, and social deficits in young children with severe TBI that can adversely impact skill development and participation in later stages of childhood into adulthood.^29,30^ It may be beneficial for all younger children with severe TBI to be referred for early childhood intervention services when skill development is below expected levels for age, as rehabilitation services are known to improve outcomes of children with severe TBI, including those injured by abuse.^31^

Children with mild, cmild, and moderate injury in our cohort stayed on their developmental track, although a slightly higher proportion of children with mild TBI were identified as needing further assessment at 36 months for fine motor and problem-solving skills compared with children with OI. Small differences in the outcomes of mild TBI have been found in other studies. Crowe et al^11^ reported that children with mild TBI had average full-scale IQs at 3.5 and 7 years postinjury, but their scores were below the typically developing comparison group, who had above-average IQ scores. This study’s interpretation is limited by lack of a preinjury measure, an injured control group, and adjustment for family factors in the analysis, all of which are important in TBI outcomes.^32^

Behavioral outcomes may show different patterns of deficit and recovery than cognitive outcomes. Gagner et al^13^ studied externalizing and internalizing behaviors on the Child Behavior Checklist following mild TBI in a large cohort of children injured between ages 18 and 60 months using both an OI and a typically developing comparison group. While all of the children had mean behavioral scores within the typical range, a larger proportion of children with mild TBI had scores in the clinical or borderline range in internalizing and externalizing symptoms compared with the OI and typically developing comparison groups at 6 and 18 months postinjury, which resolved by 30 months.

The small differences found across varied outcomes in well-controlled studies may show selective vulnerability to mild TBI in some children or selective vulnerability in some skill areas. However, attribution of these differences to the mild TBI is difficult. There may be a mixture of unmeasured confounders in children’s physical or social environments, differences in baseline potential, selective neurologic vulnerability, or a constellation of these factors that would account for these small differences. Certainly, developmental follow-up of very young children with mild TBI is essential; however, it is reassuring that most children do well.

### Strengths and Limitations

This study has several strengths. These include the large sample size, allowing for separation of children with abusive and accidental injury; injury controls; the longitudinal study design; and careful assessment of TBI severity, preinjury functioning, socioeconomic status, and the family environment.

This study also has limitations. We used only parents’ report, which may differ from more objective standardized assessments. The ASQ-3 is not a diagnostic tool; rather, it is a surveillance tool designed to ensure that children are evaluated if not proceeding with typical development. In addition, we had a small number of children with moderate TBI, so we were unable to assess them as a group.

## Conclusions

In this cohort study, infants and toddlers with severe TBI were noted to have multiple deficits that persist and may worsen as children age. These findings suggest that there is a need for monitoring and targeted referral to early childhood programs and educational services through public school systems for children aged 3 years and older. However, very young children with mild or complicated mild and moderate TBI appear to stay on their developmental track in all domains and routine pediatric surveillance may be sufficient. Future research is needed to address whether some children with mild TBI have selective vulnerability of some skill areas.

## Notes

Supplement 1. **eFigure 1.** ASQ Outcome Scores: Unadjusted Mean ± SE Over Time by Injury Severity and Type **eFigure 2.** ASQ Outcome Scores: Unadjusted Mean ± SE Over Time by Age/Abuse Group **eTable 1.** ASQ Outcome Scores for Severe TBI: Unadjusted Mean (SD) by Age/Abuse Group **eTable 2.** ASQ Social Emotional Scores Above Threshold (Indicates Potential Problem) **eAppendix.** ASQ Outcomes: Final Model Terms for Adjusted Outcome Analyses

Supplement 2.
Data Sharing Statement
