# Supplementary material for: Longitudinal Developmental Outcomes of Infants and Toddlers With Traumatic Brain Injury
Source: JAMA Netw Open. 2023 Jan 17;6(1):e2251195. doi: 10.1001/jamanetworkopen.2022.51195 (PMC9856699; doi:10.1001/jamanetworkopen.2022.51195)
Supplement: Supplement 1. — eFigure 1. ASQ Outcome Scores: Unadjusted Mean ± SE Over Time by Injury Severity and Type eFigure 2. ASQ Outcome Scores: Unadjusted Mean ± SE Over Time by Age/Abuse Group eTable 1. ASQ Outcome Scores for Severe TBI: Unadjusted Mean (SD) by Age/Abuse Group eTable 2. ASQ Social Emotional Scores Above Threshold (Indicates Potential Problem) eAppendix. ASQ Outcomes: Final Model Terms for Adjusted Outcome Analyses [file jamanetwopen-e2251195-s001.pdf]

Supplementary Online Content

Keenan HT, Clark A, Holubkov R, Ewing-Cobbs L. Longitudinal developmental outcomes of infants and toddlers with traumatic brain injury. *JAMA Netw Open*. 2023;6(1):e2251195. doi:10.1001/jamanetworkopen.2022.51195

eFigure 1. ASQ Outcome Scores: Unadjusted Mean ± SE Over Time by Injury Severity and Type

eFigure 2. ASQ Outcome Scores: Unadjusted Mean ± SE Over Time by Age/Abuse Group

eTable 1. ASQ Outcome Scores for Severe TBI: Unadjusted Mean (SD) by Age/Abuse Group

eTable 2. ASQ Social Emotional Scores Above Threshold (Indicates Potential Problem)

eAppendix. ASQ Outcomes: Final Model Terms for Adjusted Outcome Analyses

This supplementary material has been provided by the authors to give readers additional information about their work.

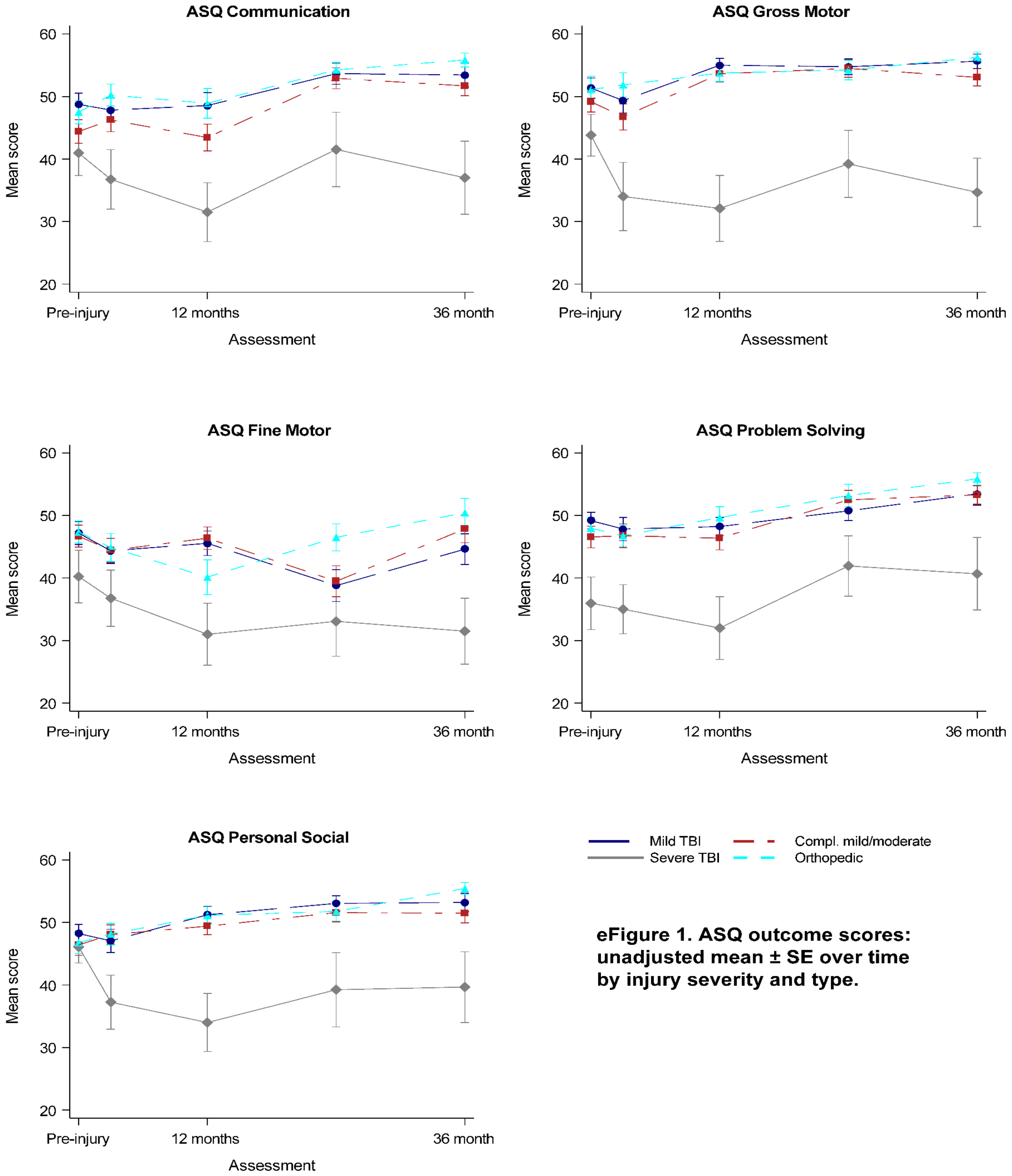

eFigure 1. ASQ outcome scores: unadjusted mean ± SE over time by injury severity and type.

© 2023 Keenan HT et al. *JAMA Network Open.*

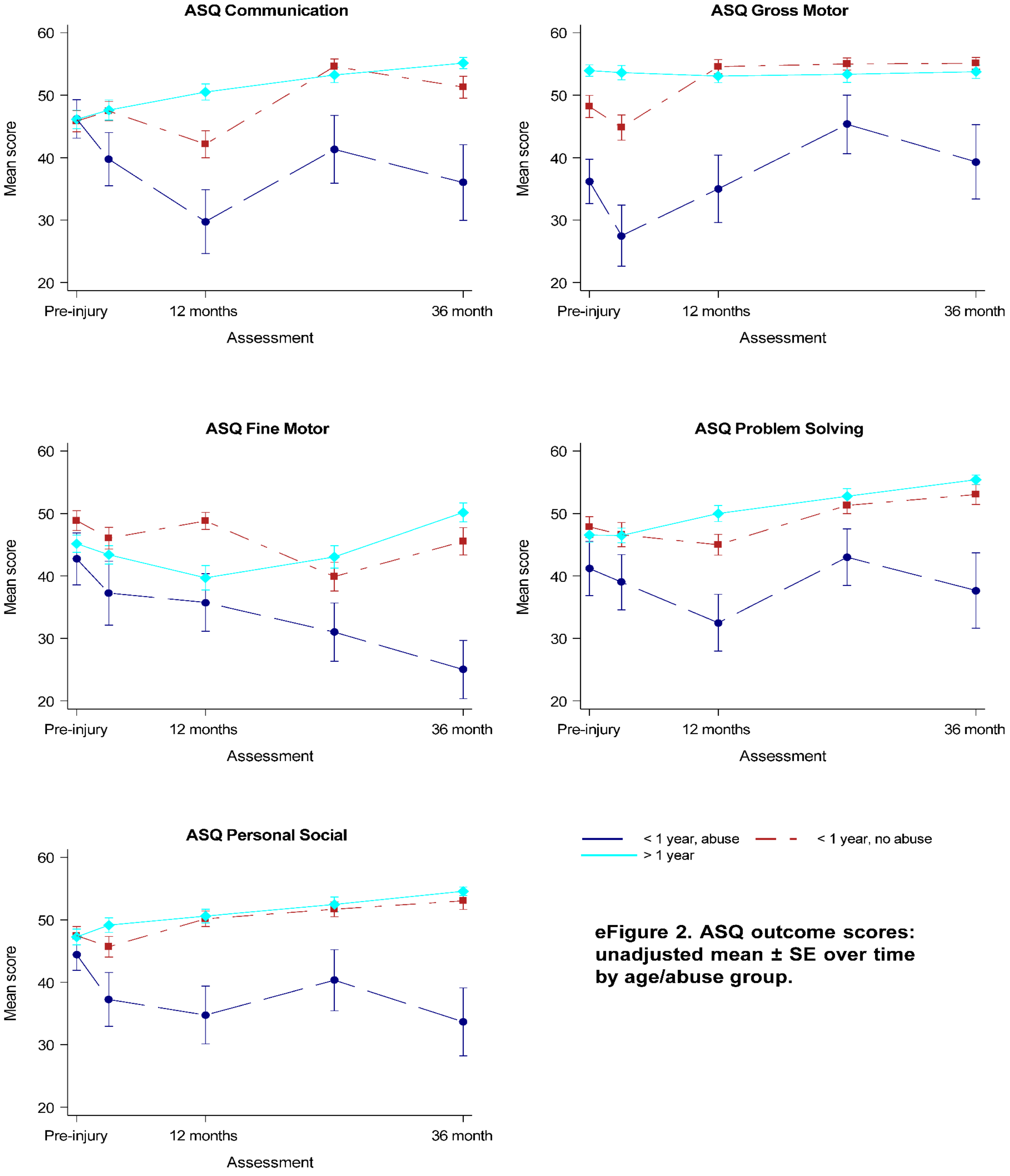

eFigure 2. ASQ outcome scores: unadjusted mean ± SE over time by age/abuse group.

© 2023 Keenan HT et al. *JAMA Network Open.*

eTable 1. ASQ outcome scores for Severe TBI: unadjusted mean (SD) by age/abuse group

	Assessment				
	Pre-injury	3 month	12 month	24 month	36 month
**Age < 1 year, abusive injury**	n=11	n=11	n=11	n=7	n=8
Communication	42.7 (14.0)	35.9 (20.6)	24.5 (23.2)	36.4 (25.9)	28.1 (26.2)
Gross motor	33.2 (13.1)	22.3 (22.1)	21.8 (24.0)	39.3 (20.5)	29.4 (24.8)
Fine motor	33.6 (22.0)	29.5 (23.0)	29.1 (24.2)	37.1 (20.6)	23.8 (21.5)
Problem solving	29.1 (20.6)	30.5 (20.1)	24.1 (23.5)	36.4 (21.9)	30.6 (26.8)
Personal-social	40.3 (11.1)	30.0 (20.7)	29.1 (24.6)	37.9 (23.4)	28.8 (25.5)
**Age < 1 year, no abusive injury**	n=2	n=2	n=2	n=1	n=1
Communication	35.0 (21.2)	40.0 (14.1)	35.0 (7.1)	60.0 (-)	55.0 (-)
Gross motor	52.5 (10.6)	45.0 (21.2)	47.5 (17.7)	40.0 (-)	50.0 (-)
Fine motor	55.0 (7.1)	57.5 (3.5)	50.0 (7.1)	15.0 (-)	40.0 (-)
Problem solving	42.5 (24.7)	45.0 (21.2)	45.0 (0.0)	55.0 (-)	60.0 (-)
Personal-social	52.5 (10.6)	40.0 (21.2)	37.5 (3.5)	30.0 (-)	50.0 (-)
**Age over 1 year**	n=8	n=7	n=7	n=5	n=6
Communication	40.0 (20.4)	37.1 (26.1)	41.4 (17.0)	45.0 (15.0)	45.8 (13.9)
Gross motor	56.3 (5.8)	49.3 (20.9)	43.9 (18.1)	39.0 (22.2)	39.2 (16.6)
Fine motor	45.6 (13.7)	42.1 (11.1)	28.6 (21.2)	31.0 (21.6)	40.4 (17.6)
Problem solving	43.8 (14.6)	39.3 (11.7)	40.7 (20.3)	47.0 (7.6)	50.8 (7.4)
Personal-social	52.5 (9.6)	47.9 (12.5)	40.7 (15.9)	43.0 (22.5)	52.5 (5.2)

ASQ: Ages & Stages Questionnaire; SD: standard deviation

© 2023 Keenan HT et al. *JAMA Network Open.*

eTable 2. ASQ Social Emotional scores above threshold (indicates potential problem)

	Injury group				
**ASQ Social Emotional score above cutoff**	**Mild TBI (N = 48)**	**Complicated Mild/Moderate (N = 54)**	**Severe TBI (N = 21)**	**Orthopedic (N = 45)**	**Overall (N = 168)**
	n (%)	n (%)	n (%)	n (%)	n (%)
**Pre-injury, n=167**	1 (2)	6 (11)	2 (10)	3 (7)	12 (7)
**3 month follow up, n=160**	2 (4)	5 (10)	7 (37)	4 (9)	18 (11)
**1 year follow up, n=160**	7 (16)	7 (14)	8 (40)	8 (18)	30 (19)
**2 year follow up, n=142**	2 (5)	10 (21)	4 (31)	5 (13)	21 (15)
**3 year follow up, n=130**	3 (8)	5 (11)	7 (54)	4 (12)	19 (15)
**New problem reported at 3 years, n=129**	3/39 (8)	2/43 (5)	6/13 (46)	2/34 (6)	13/129 (10)
**(p=0.001 for association with injury group[a])**					

ASQ: Ages & Stages Questionnaire

[a] New problem defined as "yes" if individual was above ASQ Social Emotional cutoff at 3 year follow up and below this cutoff at pre-injury. Fisher's exact test was used to evaluate the association between new problem and injury severity.

© 2023 Keenan HT et al. *JAMA Network Open.*

eAppendix. ASQ Outcomes: Final model terms for adjusted outcome analyses

Final model terms for each outcome model are specified below. All models utilized an unstructured covariance matrix for the four follow up time points (3, 12, 24 and 36 months); elements of this covariance matrix were estimated separately for the three age/abuse categories.

- **ASQ Communication:** intercept, time, $time^2$, $time^3$, pre-injury ASQ Communication, pre-injury ASQ*time, injury type, child sex, premature, age/abuse, age/abuse*time, age/abuse*$time^2$, age/abuse*$time^3$, family function, social capital.
- **ASQ Gross Motor:** intercept, time, $time^2$, $time^3$, pre-injury ASQ Gross Motor, pre-injury ASQ*time, injury type, child sex, premature, age/abuse, age/abuse*time, age/abuse*$time^2$, age/abuse*$time^3$, family function, social capital, social capital*time, social capital*$time^2$, social capital*$time^3$.
- **ASQ Fine Motor:** intercept, time, $time^2$, $time^3$, pre-injury ASQ Fine Motor, pre-injury ASQ*time, pre-injury ASQ*$time^2$, pre-injury ASQ*$time^3$, injury type, child sex, premature, age/abuse, age/abuse*time, age/abuse*$time^2$, age/abuse*$time^3$, family function, social capital.
- **ASQ Problem Solving:** intercept, time, $time^2$, $time^3$, pre-injury ASQ Problem Solving, pre-injury ASQ*time, pre-injury ASQ*$time^2$, pre-injury ASQ*$time^3$, injury type, child sex, premature, premature*time, premature*$time^2$, age/abuse, age/abuse*time, age/abuse*$time^2$, age/abuse*$time^3$, family function, social capital.
- **ASQ Personal Social:** intercept, time, $time^2$, $time^3$, pre-injury ASQ Personal Social, pre-injury ASQ*time, pre-injury ASQ*$time^2$, injury type, child sex, premature, premature*time, age/abuse, age/abuse*time, family function, social capital.

© 2023 Keenan HT et al. *JAMA Network Open.*
