# Supplementary material for: Longitudinal Developmental Outcomes of Infants and Toddlers With Traumatic Brain Injury
Source: JAMA Netw Open. 2023 Jan 17;6(1):e2251195. doi: 10.1001/jamanetworkopen.2022.51195 (PMC9856699; doi:10.1001/jamanetworkopen.2022.51195)
Supplement: Supplement 2. — Data Sharing Statement [file jamanetwopen-e2251195-s002.pdf]

Data Sharing Statement

Keenan. Longitudinal Developmental Outcomes of Infants and Toddlers With Traumatic Brain Injury. *JAMA Netw Open*. Published January 17, 2023.
doi:10.1001/jamanetworkopen.2022.51195

Data
Data available: No

Additional Information
Explanation for why data not available: De-identifed data will be made available after documentation as appropriate is completed by the relevant entities.
